# The effects of cholesterol lowering with simvastatin on cause-specific mortality and on cancer incidence in 20,536 high-risk people: a randomised placebo-controlled trial [ISRCTN48489393]

**DOI:** 10.1186/1741-7015-3-6

**Published:** 2005-03-16

**Authors:** Heart Protection Study Collaborative Group

**Affiliations:** 1 Heart Protection Study, Clinical Trial Service Unit & Epidemiological Studies Unit, Harkness Building, Radcliffe Infirmary, Oxford OX2 6HE, UK

## Abstract

**Background:**

There have been concerns that low blood cholesterol concentrations may cause non-vascular mortality and morbidity. Randomisation of large numbers of people to receive a large, and prolonged, reduction in cholesterol concentrations provides an opportunity to address such concerns reliably.

**Methods:**

20,536 UK adults (aged 40–80 years) with vascular disease or diabetes were randomly allocated to receive 40 mg simvastatin daily or matching placebo. Prespecified safety analyses were of cause-specific mortality, and of total and site-specific cancer incidence. Comparisons between all simvastatin-allocated versus all placebo-allocated participants (ie, "intention-to-treat") involved an average difference in blood total cholesterol concentration of 1.2 mmol/L (46 mg/dL) during the scheduled 5-year treatment period.

**Results:**

There was a highly significant 17% (95% CI 9–25) proportional reduction in vascular deaths, along with a non-significant reduction in all non-vascular deaths, which translated into a significant reduction in all-cause mortality (p = 0.0003). The proportional reduction in the vascular mortality rate was about one-sixth in each subcategory of participant studied, including: men and women; under and over 70 years at entry; and total cholesterol below 5.0 mmol/L or LDL cholesterol below 3.0 mmol/L. No significant excess of non-vascular mortality was observed in any subcategory of participant (including the elderly and those with pretreatment total cholesterol below 5.0 mmol/L), and there was no significant excess in any particular cause of non-vascular mortality.

Cancer incidence rates were similar in the two groups, both overall and in particular subcategories of participant, as well as at particular primary sites. There was no suggestion that any adverse trends in non-vascular mortality or morbidity were beginning to emerge with more prolonged treatment.

**Conclusion:**

These findings, which are based on large numbers of deaths and non-fatal cancers, provide considerable reassurance that lowering total cholesterol concentrations by more than 1 mmol/L for an average of 5 years does not produce adverse effects on non-vascular mortality or cancer incidence. Moreover, among the many different types of high-risk individual studied, simvastatin 40 mg daily consistently produced substantial reductions in vascular (and, hence, all-cause) mortality, as well as in the rates of non-fatal heart attacks, strokes and revascularisation procedures.

## Background

Observational studies have found blood total cholesterol concentrations below about 4.0 mmol/L (155 mg/dL) to be associated with higher rates of mortality and morbidity from certain non-vascular causes (in particular, cancer of lung, liver, pancreas and blood; chronic obstructive pulmonary disease; cirrhosis; suicide; and haemorrhagic stroke) [1,2]. Excluding events within the first few years after the baseline measurement of cholesterol attenuates some, but not all, of these associations. It remains unclear, however, whether these inverse associations are causal (with low cholesterol actually causing certain diseases), or instead due to confounding or reverse causation (with certain habits or conditions independently causing both lower cholesterol and disease) [3,4]. Randomised trials are not subject to such biases, but the trials of cholesterol-lowering interventions before the statins were unable to assess causation reliably, chiefly because they involved too few non-vascular outcomes (even in combination) and assessed only modest cholesterol reductions from high pretreatment concentrations [5,6]. Some slight excesses of non-vascular deaths or cancers in particular trials provoked much comment over the years, contributing to the uncertainty that surrounded cholesterol-lowering [7-11].

More recently, in previous trials of statin treatment, LDL cholesterol was typically reduced by about 1.0 mmol/L (38 mg/dl) and major vascular events reduced by about 25% [12-19]. Although meta-analyses of those trials indicate that statin therapy reduces vascular mortality [20-23], the secondary prevention trials involved few deaths from non-vascular causes [12-15], and the primary prevention trials involved relatively few deaths from any cause [16-19]. Moreover, these trials provided relatively little information about the effects of lowering total cholesterol to the low concentrations (e.g. around 4.0 mmol/L) that had previously raised concerns. By contrast, the Heart Protection Study (HPS) involved large numbers of deaths from both vascular and non-vascular causes among people presenting with below-average cholesterol levels who were randomly allocated to have their cholesterol lowered substantially for several years [24]. Consequently, it allows outstanding concerns about the potential hazards of lowering cholesterol to be addressed much more reliably than has previously been possible. The present report provides detailed information about the effects of lowering cholesterol on cause-specific mortality, site-specific cancer incidence and other major morbidity in a range of different circumstances.

## Methods

Details of HPS have been reported previously [24-29] (see also ) and are summarised below.

### Recruitment and follow-up

Men and women aged about 40 to 80 years with non-fasting blood total cholesterol concentrations of at least 3.5 mmol/L (135 mg/dL) were eligible provided they had a medical history of: occlusive arterial disease; diabetes mellitus; or treated hypertension (if also male and aged at least 65 years). People were ineligible if their own doctor considered statin therapy to be clearly indicated or contraindicated, or if they had a past history of: stroke, myocardial infarction or angina hospitalisation within the previous 6 months; chronic liver disease or evidence of abnormal liver function; severe renal disease or evidence of substantially impaired renal function; inflammatory muscle disease or evidence of muscle problems; concurrent treatment with cyclosporin, fibrates or high-dose niacin; child-bearing potential; severe heart failure; life-threatening conditions other than vascular disease or diabetes (including any cancer except non-melanoma skin cancer); or any other condition that might limit long-term compliance.

Eligible and consenting patients entered a pre-randomisation "run-in" treatment phase, which involved 4 weeks of placebo followed by 4–6 weeks of 40 mg simvastatin daily. Compliant individuals who were still not considered by their own doctors to have a clear indication for, or contraindication to, statin therapy were then randomly allocated to receive 40 mg simvastatin daily or matching placebo tablets for about 5 years (and separately, using a 2 × 2 factorial design, to receive antioxidant vitamins or matching placebo capsules [29]). Randomised participants were to be seen at 4, 8 and 12 months, and then 6-monthly (with follow-up by telephone for individuals who did not attend or, alternatively, via their general practitioner). Compliance with study treatment was assessed by questioning participants and reviewing the calendar-packed tablets remaining. Blood samples were taken at each follow-up visit for central laboratory assay of alanine transaminase to monitor liver function, and of creatine kinase in any participant reporting unexplained muscle symptoms or concomitant use of a non-study statin. To assess the effects of the treatment allocation on the lipid profile, assays were performed on non-fasting blood collected from a sample of participants due for follow-up at about the same time each year, and from all participants attending follow-up at an average of 4.6 years. Differences in average blood lipid concentrations were based on comparisons between all those allocated simvastatin and all those allocated placebo, irrespective of whether or not they were still compliant.

Information was recorded at each follow-up about any suspected myocardial infarction, stroke, vascular procedure, cancer or other serious adverse experience, and about the main reasons for all other hospital admissions. UK national registries provided information about the sites of any registered cancers and the certified causes of death. Further details were sought from general practitioners (and, if considered necessary, hospital records) about all reports that might relate to major vascular events, cancers (e.g. investigations) or deaths. All such information was coded according to prespecified criteria by coordinating centre clinical staff, who were unaware of the participants' study treatment allocation. Analyses were based on confirmed plus unrefuted reports of events. Cancers were classified according to their primary anatomical site rather than their histology (except that skin cancers were subclassified as melanoma or non-melanoma), with definite confirmation for 98% of the included cancers. Mortality follow-up was available for 99.7% of participants, with certified causes for 98.2% of deaths.

### Statistical analyses

The main comparisons involved logrank analyses of the first occurrence of particular events during the scheduled treatment period among all those allocated simvastatin versus all those allocated matching placebo tablets (i.e. "intention-to-treat"). These logrank analyses yielded both the event rate ratio (RR) and the test of statistical significance (two-sided probability value). The prespecified primary comparisons were of the effects of allocation to simvastatin on deaths from all causes and, separately, on deaths from all coronary causes and from all non-coronary causes. But since simvastatin appeared to reduce the risk of death not only from coronary causes but also from other vascular causes (as well as preventing non-fatal vascular events) [24], the present analyses are of all vascular deaths and of all non-vascular deaths.

Prespecified secondary comparisons included the effects of simvastatin allocation on specific vascular and non-vascular causes of death [25], with due allowance made in their interpretation for multiple hypothesis testing, for the effects observed on relevant non-fatal events, and for evidence from other studies. It was not anticipated that there would be adequate statistical power to assess the effects of study treatment on vascular or total mortality directly in different circumstances. Instead, the prespecified comparisons involved assessment of the effects on major coronary events (defined as non-fatal myocardial infarction or coronary death), and on the even larger numbers of major vascular events (i.e. major coronary event, stroke or arterial revascularisation), in the first two years and in the later years of scheduled treatment (in order to determine whether any protective effect increases with time), and in various prespecified subcategories determined at study entry, including: sex; age (<65; ≥65<70; ≥70 years); and pretreatment plasma concentrations of total cholesterol (<5.0; ≥5.0<6.0; ≥6.0 mmol/L) and of LDL cholesterol (<3.0; ≥3.0<3.5; ≥3.5 mmol/L). Pre-specified tertiary comparisons included the effects on vascular mortality separately during years 1–2 and years 3+ of follow-up, on cause-specific mortality in the prespecified subcategories of pretreatment total cholesterol, and on site-specific cancer incidence and cerebral haemorrhage. Tests for heterogeneity or, if more appropriate, trend were to be used to assess whether the proportional effects observed on particular outcomes in specific subcategories differed clearly from the overall effect (after due allowance for multiple comparisons).

### Role of the funding sources

The investigators were responsible for the study design, data collection, data analysis, data interpretation, and writing of the report, independently of all funding sources.

## Results

### Patient enrolment

A total of 20,536 individuals were randomised between July 1994 and May 1997. Mean age at study entry was 64.0 years (SD 8.4), with 5806 aged at least 70 years. Prior to any statin treatment being started, participants who were subsequently randomised had mean non-fasting plasma concentrations of total cholesterol of 5.9 mmol/L (SD 1.0), directly-measured LDL cholesterol of 3.4 mmol/L (0.8), HDL cholesterol of 1.06 mmol/L (0.3) and triglycerides of 2.1 mmol/L (1.4). There were 4072 randomised participants whose pretreatment measurements of total cholesterol were below 5.0 mmol/L (193 mg/dL) and 6793 with pretreatment LDL cholesterol below 3.0 mmol/L (116 mg/dL). The large size of the study (and the use of minimised randomisation [30]) produced good balance between the treatment groups for the main prognostic features that were measured (see subcategory figures below), and should have done likewise for those that were not.

### Compliance and effects on blood lipids

The mean duration of follow-up was 5.0 years for all randomised participants: 5.3 years for those who survived to the scheduled end of study treatment and about half that for those who did not (yielding 51,121 person years of follow-up for all those allocated simvastatin and 50,664 for all those allocated placebo). Compliance at each follow-up was defined as at least 80% of the scheduled simvastatin or placebo tablets having been taken since the previous follow-up. Among the participants allocated 40 mg simvastatin daily, average statin use during the scheduled treatment period was 85% (with 82% compliant with their allocated simvastatin, 3% on non-study statin alone and 2% on both). By contrast, among those allocated placebo, 4% at the end of the first year of follow-up, but 32% at the end of the fifth year, were taking non-study statin therapy, yielding an average of 17%. Non-study statin use in the placebo group was more common among those who already had diagnosed coronary disease at entry, were younger or had higher pretreatment total or LDL cholesterol concentrations [24]. In each subcategory studied, however, the average difference in total cholesterol was about 1.2 mmol/L (range: 1.1–1.3 mmol/L: table 1) and in LDL cholesterol was about 1.0 mmol/L (range: 0.9–1.1 mmol/L). In particular, among the 4072 participants whose pretreatment measurements of total cholesterol were below 5.0 mmol/L (193 mg/dL), the average total cholesterol concentration during the trial was 3.5 mmol/L in the simvastatin group compared to 4.6 mmol/L in the placebo group.

**Table 1:** Average plasma total and LDL cholesterol concentrations during follow-up

**Baseline characteristic**	**Plasma total cholesterol (mmol/l)**			**Plasma LDL cholesterol (mmol/l)**		
	**Simvastatin**	**Placebo**	Difference*	**Simvastatin**	**Placebo**	Difference*
						
**Sex**						
Male	4.1	5.3	-1.2	2.2	3.2	-1.0
Female	4.6	5.8	-1.2	2.5	3.4	-0.9
						
**Age (years)**						
<65	4.3	5.4	-1.1	2.4	3.2	-0.9
≥65 <70	4.1	5.4	-1.3	2.2	3.3	-1.0
≥70	4.2	5.5	-1.3	2.2	3.3	-1.1
						
**Total cholesterol (mmol/L)**						
<5.0	3.5	4.6	-1.1	1.8	2.6	-0.9
≥5.0 <6.0	4.0	5.2	-1.2	2.1	3.1	-1.0
≥6.0	4.8	6.0	-1.2	2.7	3.7	-1.0
						
**LDL cholesterol (mmol/L)**						
<3.0	3.7	4.9	-1.1	1.8	2.7	-0.9
≥3.0 <3.5	4.1	5.3	-1.2	2.2	3.2	-1.0
≥3.5	4.7	5.9	-1.2	2.7	3.7	-1.0
						
**ALL PATIENTS**	**4.2**	**5.4**	**-1.2**	**2.3**	**3.3**	**-1.0**

* Intention-to-treat comparisons with missing data imputed from initial pre-treatment screening values. The absolute difference in LDL-cholesterol that would be produced by full compliance with 40 mg simvastatin daily can be estimated as the ratio of the absolute difference to the estimated compliance. For example, -1.0/67% = -1.5 mmol/L.

### Effects on vascular mortality and morbidity

Overall, allocation to simvastatin produced a highly significant 17% (SE 4; p < 0.0001) proportional reduction in vascular mortality during the 5 years of the study (table 2). This reflected a definite 18% (SE 5; p = 0.0005) reduction in deaths due to coronary causes, together with a non-significant 20% (SE 12; p = 0.1) reduction in fatal strokes and 12% (SE 13; p = 0.3) reduction in deaths from other vascular causes, with no significant difference between the effects observed on these different vascular causes (heterogeneity p = 0.9). Among the coronary causes there were also no statistically significant differences among the effects of statin allocation on deaths attributed to acute myocardial infarction, sudden death, heart failure secondary to coronary disease, or other coronary causes (heterogeneity p = 0.5). There was no apparent difference between the treatment groups in the small number of deaths attributed to cerebral haemorrhage, whereas there was a non-significant reduction in fatal strokes due to ischaemic (or unknown) causes. Other vascular causes comprised deaths from peripheral vascular disease (which included ruptured aortic aneurysm) and from a variety of other cardiac conditions (including a few attributed to heart failure), again with no significant heterogeneity in the effects of simvastatin allocation on these different causes of death (heterogeneity p = 0.7).

**Table 2:** Effect of simvastatin allocation on vascular and non-vascular causes of death

**Cause of death**	**Simvastatin-allocated (10,269)**	**Placebo- allocated (10,267)**	**Death rate ratio (& 95% CI)**	**P-value**
				
**Coronary**				
Acute MI	141 (1.4%)	191 (1.9%)	0.73 (0.59 – 0.91)	
Sudden death	147 (1.4%)	154 (1.5%)	0.95 (0.75 – 1.19)	
Heart failure*	65 (0.6%)	78 (0.8%)	0.82 (0.59 – 1.15)	
Other coronary	234 (2.3%)	284 (2.8%)	0.82 (0.69 – 0.97)	
**Subtotal: Coronary**	**587 (5.7%)**	**707 (6.9%)**	**0.82 (0.74 – 0.92)**	**0.0005**
				
**Stroke**				
Haemorrhagic	23 (0.2%)	24 (0.2%)	0.95 (0.54 – 1.68)	
Ischaemic (or unknown)	73 (0.7%)	95 (0.9%)	0.76 (0.56 – 1.03)	
**Subtotal: Stroke**	**96 (0.9%)**	**119 (1.2%)**	**0.80 (0.61 – 1.05)**	**0.1**
				
**Other vascular**				
Peripheral vascular	58 (0.6%)	63 (0.6%)	0.91 (0.64 – 1.30)	
Other cardiac*	40 (0.4%)	48 (0.5%)	0.83 (0.54 – 1.25)	
**Subtotal: Other vascular**	**98 (1.0%)**	**111 (1.1%)**	**0.88 (0.67 – 1.15)**	**0.3**
				
**VASCULAR**	**781 (7.6%)**	**937 (9.1%)**	**0.83 (0.75 – 0.91)**	**<0.0001**
				
**Neoplastic**				
Respiratory	127 (1.2%)	133 (1.3%)	0.94 (0.74 – 1.20)	
Gastrointestinal	112 (1.1%)	103 (1.0%)	1.08 (0.82 – 1.41)	
Genitourinary	47 (0.5%)	46 (0.4%)	1.01 (0.67 – 1.52)	
All others	73 (0.7%)	62 (0.6%)	1.17 (0.83 – 1.63)	
**Subtotal: Neoplastic**	**359 (3.5%)**	**345 (3.4%)**	**1.03 (0.89 – 1.19)**	**0.7**
				
**Other non-vascular**				
Respiratory	90 (0.9%)	114 (1.1%)	0.78 (0.59 – 1.03)	
Gastrointestinal	35 (0.3%)	41 (0.4%)	0.85 (0.54 – 1.33)	
Other medical^†^	47 (0.5%)	49 (0.5%)	0.95 (0.64 – 1.42)	
Non-medical	16 (0.2%)	21 (0.2%)	0.75 (0.40 – 1.44)	
				
**NON-VASCULAR**	**547 (5.3%)**	**570 (5.6%)**	**0.95 (0.85 – 1.07)**	**0.4**
				
**ALL DEATHS**	**1328 (12.9%)**	**1507 (14.7%)**	**0.87 (0.81 – 0.94)**	**0.0003**

* Heart failure deaths were subdivided into those considered to be due to coronary disease and those (5 versus 8) due to other vascular (or unknown) causes.

† Includes renal, infectious, metabolic, neurological and unknown deaths.

**Figure 1:**
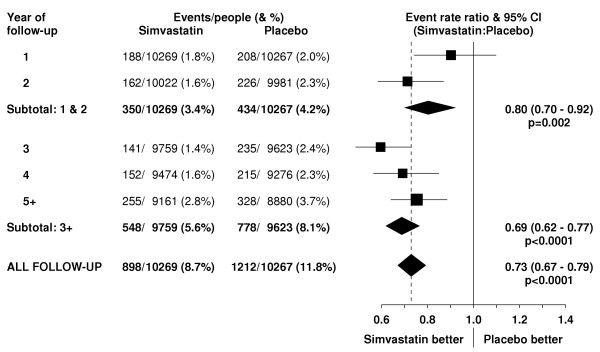
**Effect of simvastatin allocation on major coronary events by year of follow-up**. Rate ratios (RR: black squares with area proportional to the amount of "statistical information" in each subdivision) comparing outcome among patients allocated simvastatin to that among those allocated placebo are plotted, along with their 95% confidence intervals (CI: horizontal lines). For relevant subtotals, the result and its 95% CI is represented by a diamond, with the overall proportional reduction and statistical significance given alongside. Squares or diamonds to the left of the solid vertical line indicate benefit with simvastatin (with nominal significance of at least two-sided P < 0.05 when the horizontal line or diamond does not overlap the vertical line). The broken vertical lines indicate the overall rate ratios.

The apparent reductions in different types of vascular death with allocation to simvastatin are reinforced by more definite effects on the larger numbers of non-fatal and fatal vascular events considered together. For example, the very definite 27% (SE 4; p < 0.0001: figure 1) reduction in first non-fatal myocardial infarction or coronary death (i.e. "major coronary events") following randomisation reinforces the observed effect on coronary death [24]. Similarly, the definite 25% (SE 5; p < 0.0001) reduction in first non-fatal or fatal stroke following randomisation (444 [4.3%] simvastatin versus 585 [5.7%] placebo) indicates that the trend toward fewer fatal strokes with simvastatin is likely to be real [28]. Despite concerns about possible adverse effects of statin therapy on heart failure [31], the non-significant trend toward fewer heart failure deaths due to any cause (70 [0.7%] vs 86 [0.8%]; RR 0.81 [0.59–1.10]; p = 0.2) is supported by a marginally significant reduction in first hospital admission for worsening heart failure or heart failure death (354 [3.4%] vs 405 [3.9%]; RR 0.86 [0.75–1.00]; p = 0.05).

**Figure 2:**
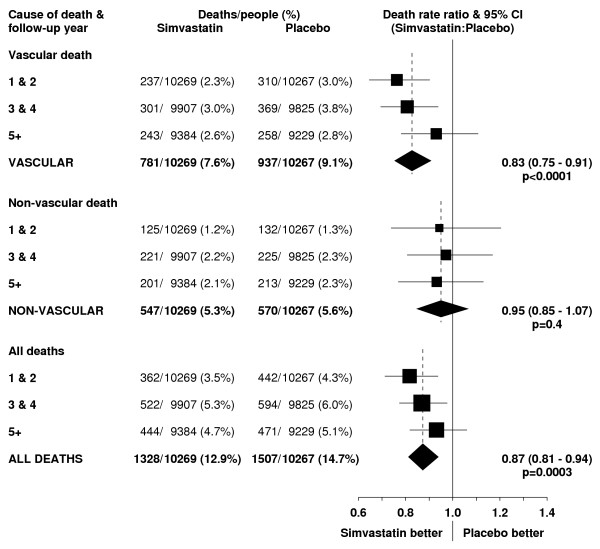
**Effect of simvastatin allocation on vascular, non-vascular and all-cause mortality by period of follow-up**. Symbols and conventions as in Figure 1.

**Figure 3:**
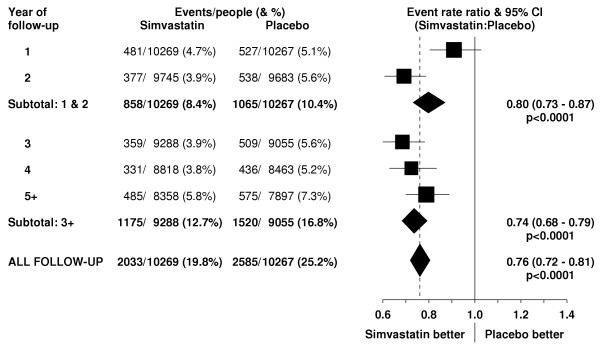
**Effect of simvastatin allocation on major vascular events by year of follow-up**. Symbols and conventions as in Figure 1.

**Figure 4:**
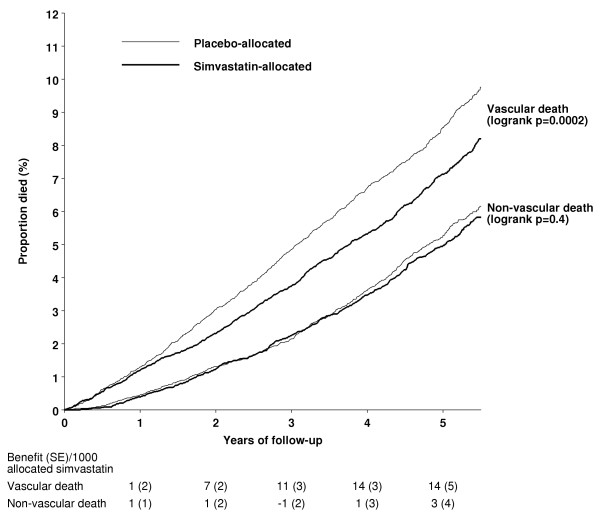
**Life-table plot of effects of simvastatin allocation on vascular and non-vascular death**. See figure 2 for numbers of participants dying during follow-up.

In this high-risk population with occlusive arterial disease or diabetes, about 1.5% of placebo-allocated patients died from vascular causes during each year of follow-up. A highly significant 24% (95% CI 10–35; p = 0.002) proportional reduction in vascular mortality emerged during the first two years after the initiation of simvastatin treatment (figure 2), which is reinforced by the prespecified analyses of major coronary events (figure 1) and of major vascular events (figure 3). Further reductions in vascular mortality were observed during the subsequent years of the scheduled treatment period, and again these are reinforced by the observed effects on vascular events. About one third of the placebo-allocated participants were taking a statin by the end of year 5, and this may account for the somewhat smaller reductions in vascular deaths (and vascular events) observed during the later years. Even so, the continuing reductions in vascular mortality (and morbidity) during each period resulted in increasing absolute benefits with more prolonged treatment and follow-up (figure 4). As a consequence, whereas 9.1% of the placebo-allocated patients died of vascular causes during an average of 5 years of follow-up, only 7.6% of those allocated simvastatin did so. Hence, lowering LDL cholesterol by an average of 1.0 mmol/L for 5 years was associated with the prevention of 14 (SE 5) vascular deaths per 1000 participants.

**Figure 5:**
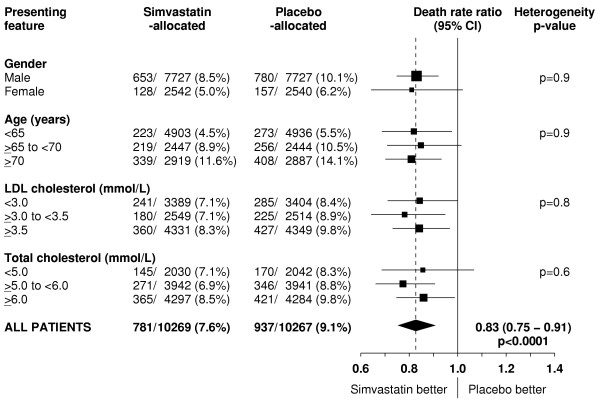
**Effect of simvastatin allocation on vascular death in participants subdivided by presenting features**. Symbols and conventions as in Figure 1. P-values for chi-squared tests for heterogeneity across different subgroups are given on the right.

**Figure 6:**
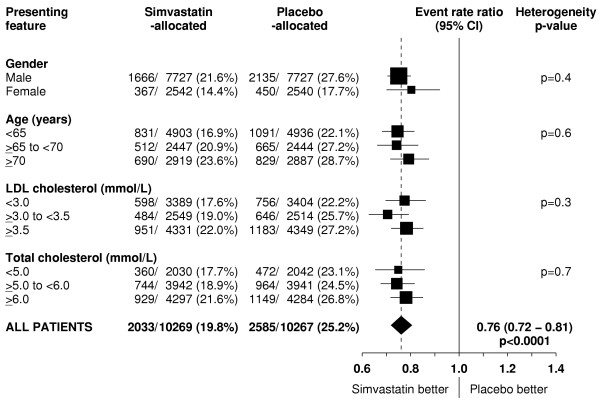
**Effect of simvastatin allocation on first major vascular event in participants subdivided by presenting features**. Symbols and conventions as in Figures 1 & 5.

Figure 5 indicates that the proportional reduction in the rate of vascular death was about one sixth in various different circumstances, and this pattern is also reinforced by the prespecified subgroup analyses of the much larger numbers of major vascular events (figure 6). For example, there was a 17% (SE 5; p = 0.0004) reduction in vascular mortality among the 15,454 men and a 19% (SE 11; p = 0.08) reduction among the 5082 women (heterogeneity p = 0.9 between effect in men vs women), which is reinforced by the highly significant reductions in major vascular events both among men and among women. Similarly, the proportional reductions in vascular mortality appeared to be about the same among younger and older participants (heterogeneity p = 0.9), as was also the case for major vascular events. But since the older participants were at higher absolute risk of vascular death, these similar proportional effects translated into larger absolute benefits at older ages during the 5 year treatment period. The reduction in vascular mortality also appeared largely independent of the pretreatment lipid concentrations, and this pattern is again reinforced by the parallel analyses of major vascular events.

### Effects on non-vascular mortality and morbidity

Overall, there was no evidence that reducing total cholesterol by an average of 1.2 mmol/L for 5 years produced any adverse effect on the aggregate of all non-vascular deaths (RR 0.95 [95% CI 0.85–1.07]; p = 0.4: table 2). More than half of these non-vascular deaths were due to cancer, and there were no significant differences between the treatment groups in the numbers of deaths from all cancers or from cancers at particular sites (or in the numbers of incident cancers: see below). Among participants allocated simvastatin there were non-significantly fewer deaths from all respiratory causes, including those due to chronic obstructive pulmonary disease (26 [0.3%] simvastatin vs 39 [0.4%] placebo; RR 0.66 [0.41–1.08]; p = 0.1). This apparent lack of any adverse effect on respiratory death is reinforced by the similar numbers of participants in the two treatment groups who either died from, or were admitted to hospital for, any respiratory illness (811 [7.9%] simvastatin vs 820 [8.0%] placebo; RR 0.98 [0.89–1.08]; p = 0.7) or for chronic obstructive pulmonary disease (88 [0.9%] vs 110 [1.1%]; RR 0.79 [0.60–1.05]; p = 0.1). Moreover, lung function assessed by spirometry in all those attending the final visit showed no differences between the treatment groups: forced expiratory volume in 1 second (FEV_1_) of 2.06 L simvastatin vs 2.05 L placebo (difference 0.01 L [SE 0.01]; p = 0.5); and forced vital capacity (FVC) of 2.82 L vs 2.82 L (difference 0.00 L [SE 0.01]; p = 0.9). This was the case even among participants with pretreatment total cholesterol measurements below 5.0 mmol/L: FEV_1 _of 2.16 L vs 2.15 L (difference 0.00 L [SE 0.03]; p = 0.9); and FVC of 2.94 L vs 2.95 L (difference 0.00 [SE 0.03]; p > 0.9).

There were similar numbers of deaths in the two treatment groups from gastrointestinal causes, which included a small number attributed to liver disease (5 simvastatin vs 3 placebo). Few patients reported developing cirrhosis (4 vs 4), but there was no apparent adverse effect on the much larger number with any non-fatal or fatal liver-related serious adverse event, either overall (197 [1.9%] vs 200 [1.9%]; RR 0.98 [0.80–1.19]; p = 0.8) or among those with pretreatment total cholesterol measurements below 5.0 mmol/L (48 [2.4%] vs 41 [2.0%]; RR 1.18 [0.78–1.78]; p = 0.4). There was also no suggestion of any adverse effect of cholesterol-lowering therapy on other medical causes of death (table 2), which included small numbers due to infections (7 vs 17) and renal disease (10 vs 10). This apparent lack of effect on renal death is reinforced by the similar numbers in the two treatment groups who developed renal failure or died from renal causes (71 [0.7%] vs 63 [0.6%]; RR 1.12 [0.80–1.57]; p = 0.5). Relatively few non-medical deaths occurred, with the majority being due to accidents and injuries (12 vs 15) and the remainder due to complications of medical or surgical procedures (4 vs 5) or suicide (0 vs 1). There was no apparent excess in the numbers who reported attempted suicide (14 [0.1%] vs 11 [0.1%]; RR 1.26 [0.58–2.76]; p = 0.6), development of depression (39 [0.4%] vs 34 [0.3%]; RR 1.14 [0.72–1.80]; p = 0.6) or any psychiatric disorder (96 [0.9%] vs 90 [0.9%]; RR 1.06 [0.79–1.41]; p = 0.7).

**Figure 7:**
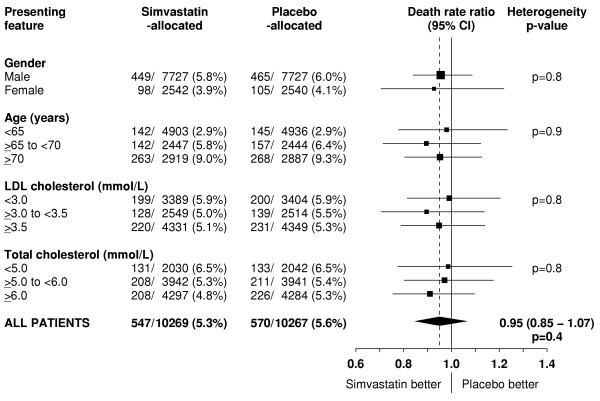
**Effect of simvastatin allocation on non-vascular death in participants subdivided by presenting features**. Symbols and conventions as in Figures 1 & 5.

Any adverse effects of lowering cholesterol might be expected to emerge only after some years of lower cholesterol levels, but there was no suggestion of an excess of non-vascular death even during the later years of the study (figures 2 and 4). For example, there were similar rates of non-vascular death in each treatment group during years 3 & 4 (RR 0.97 [0.81–1.17]; p = 0.8) and during years 5+ (RR 0.93 [0.77–1.13]; p = 0.5). Nor did lowering total cholesterol by an average of about 1.2 mmol/L for 5 years produce an excess of non-vascular mortality in any of the different types of patient studied (figure 7). For example, inverse associations between cholesterol concentrations and mortality have been reported from some observational studies among the very elderly [32,33]. But no adverse effect on non-vascular mortality was seen among the 5806 participants in HPS who were aged 70 years or older at entry (RR 0.95 [0.80–1.13]; p = 0.6) or, indeed, even among the 1263 participants who were aged 75–80 years at entry (60 [9.8%] vs 78 [12.0%]; RR 0.78 [0.56–1.09]; p = 0.1). Similarly, despite concerns from non-randomised observational studies about higher mortality in association with low cholesterol concentrations, there was no adverse effect on non-vascular mortality even among the 4072 participants with pretreatment total cholesterol measurements below 5.0 mmol/L (RR 0.99 [0.77–1.26]; p = 0.9), in whom simvastatin allocation reduced total cholesterol concentrations to an average of 3.5 mmol/L (Table 1).

### Effects on all-cause mortality

**Figure 8:**
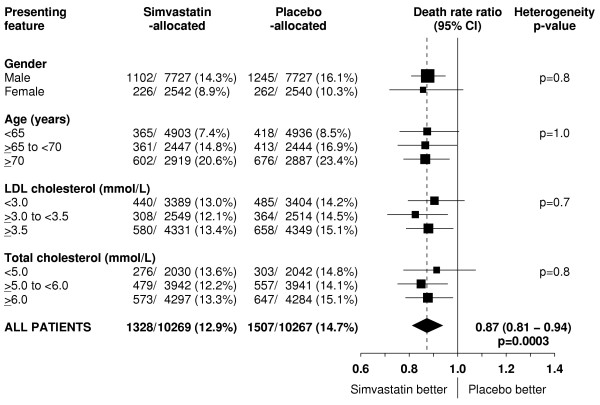
**Effect of simvastatin allocation on all-cause mortality in participants subdivided by presenting features**. Symbols and conventions as in Figures 1 & 5.

The lack of any overall effect of cholesterol-lowering therapy on non-vascular mortality in different circumstances (figure 7) suggests that the proportional reduction in all-cause mortality in any particular circumstance is likely to depend chiefly on the fraction of deaths that are due to vascular causes and on the proportional reduction in vascular mortality that is produced by lowering LDL cholesterol. For example, in the present study, the 13% proportional reduction in all-cause mortality produced by lowering LDL cholesterol by an average of about 1 mmol/L with statin therapy reflects the combination of the highly significant 17% reduction in the two-thirds of deaths due to vascular causes and the lack of any significant difference in the remaining one third of deaths from non-vascular causes (table 2). Figure 2 illustrates this combined effect on all-cause mortality over time, and the similar proportional reductions in total mortality in different types of participant (figure 8) reflect the consistent beneficial effects of simvastatin allocation on vascular death, and consistent lack of effect on non-vascular deaths, across subgroups.

### Effects on cancer incidence

**Figure 9:**
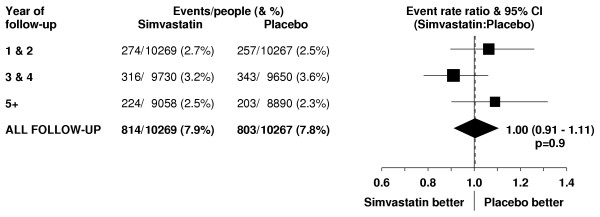
**Effect of simvastatin allocation on incidence of first cancer by period of follow-up**. Symbols and conventions as in Figure 1. Excludes non-melanoma skin cancer.

**Figure 10:**
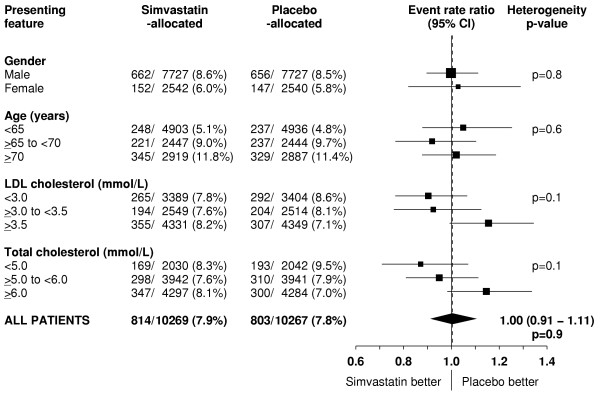
**Effect of simvastatin allocation on cancer incidence in participants subdivided by presenting features**. Symbols and conventions as in Figures 1 & 5. Excludes non-melanoma skin cancer.

Overall, there was no evidence that reducing total cholesterol from an average of 5.4 mmol/L to an average of 4.2 mmol/L (table 1) produced any adverse effect on the incidence of first cancer (excluding non-melanoma skin) at any site following randomisation (814 [7.9%] simvastatin vs 803 [7.8%] placebo; RR 1.00 [0.91–1.11]; p = 0.9: table 3). Moreover, no significant excess of these cancers emerged even during the later years of the study (figure 9). Nor was there any significant evidence that cholesterol-lowering therapy produced adverse effects on the cancer incidence rate in any particular type of participant studied (figure 10). For example, by contrast with a marginally significant adverse trend in one recently reported randomised trial of statin therapy [34], simvastatin allocation was not associated with a significant excess of cancer among the 5806 participants aged 70 years or older at presentation (RR 1.02 [0.88–1.19]; p = 0.8). Furthermore, whereas non-randomised observational studies have found higher cancer rates in association with low cholesterol levels [1,2], no adverse effect was seen on the cancer incidence rate in this large randomised comparison among the 4072 participants with pretreatment total cholesterol measurements below 5.0 mmol/L (RR 0.87 [0.71–1.07]; p = 0.2).

**Table 3:** Effect of simvastatin allocation on site-specific cancer incidence

**Cancer site**	**Simvastatin-allocated (10,269)**	**Placebo-allocated (10,267)**	**Event rate ratio (& 95% CI)**	**P-value**
				
**Respiratory**				
Lung/larynx	171 (1.7%)	157 (1.5%)	1.08 (0.87 – 1.34)	
Other	8 (0.1%)	10 (0.1%)	0.79 (0.32 – 2.00)	
**Subtotal: Respiratory**	**179 (1.7%)**	**167 (1.6%)**	**1.06 (0.86 – 1.31)**	**0.6**
				
**Gastrointestinal**				
Upper GI	74 (0.7%)	62 (0.6%)	1.18 (0.84 – 1.65)	
Colon/rectum	114 (1.1%)	131 (1.3%)	0.86 (0.67 – 1.11)	
Other	41 (0.4%)	33 (0.3%)	1.23 (0.78 – 1.94)	
**Subtotal: Gastrointestinal**	**228 (2.2%)**	**223 (2.2%)**	**1.01 (0.84 – 1.22)**	**0.9**
				
**Connective tissue**				
Female breast	38 (1.5%)	51 (2.0%)	0.74 (0.49 – 1.12)	
Melanoma	17 (0.2%)	10 (0.1%)	1.66 (0.78 – 3.54)	
Other	5 (0.0%)	7 (0.1%)	0.71 (0.23 – 2.20)	
**Subtotal: Connective tissue**	**60 (0.6%)**	**68 (0.7%)**	**0.87 (0.62 – 1.24)**	**0.4**
				
**Genitourinary**				
Renal	23 (0.2%)	22 (0.2%)	1.04 (0.58 – 1.86)	
Bladder	74 (0.7%)	90 (0.9%)	0.81 (0.60 – 1.11)	
Prostate	145 (1.9%)	145 (1.9%)	0.99 (0.79 – 1.25)	
Gynaecological	19 (0.7%)	18 (0.7%)	1.05 (0.55 – 2.00)	
Other	6 (0.1%)	6 (0.1%)	0.99 (0.32 – 3.08)	
**Subtotal: Genitourinary**	**259 (2.5%)**	**272 (2.6%)**	**0.94 (0.80 – 1.12)**	**0.5**
				
**Haematological**				
Leukaemia/lymphoma	42 (0.4%)	32 (0.3%)	1.30 (0.82 – 2.05)	
Other	23 (0.2%)	23 (0.2%)	0.99 (0.55 – 1.76)	
**Subtotal: Haematological**	**64 (0.6%)**	**52 (0.5%)**	**1.22 (0.85 – 1.75)**	**0.3**
				
**Other & unspecified**	**54 (0.5%)**	**57 (0.6%)**	**0.94 (0.65 – 1.36)**	**0.7**
				
ALL CANCERS*	**814 (7.9%)**	**803 (7.8%)**	**1.00 (0.91 – 1.11)**	**0.9**

* Prespecified that analyses of cancer incidence were to exclude non-melanoma skin cancer (243 [2.4%] simvastatin-allocated versus 202 [2.0%] placebo-allocated; p = 0.06).

**Figure 11:**
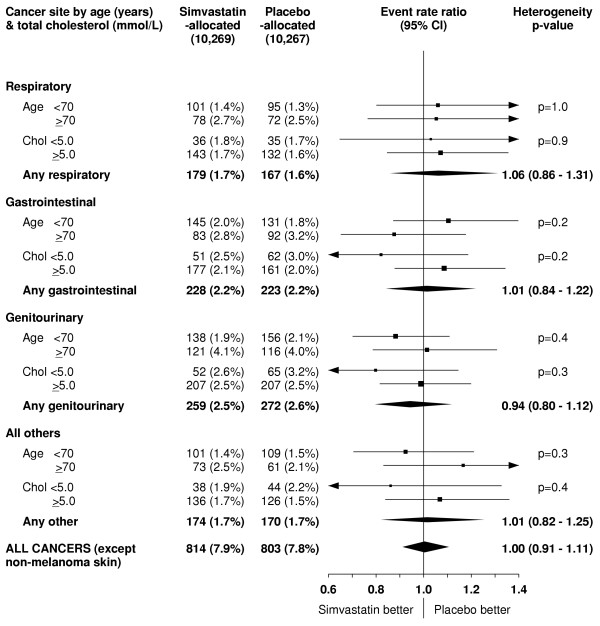
**Effect of simvastatin allocation on site-specific cancers subdivided by age and total cholesterol at study entry**. Symbols and conventions as in Figures 1 & 5. Excludes non-melanoma skin cancer.

Any adverse effects of cholesterol lowering on cancer might be expected to be restricted to cancers at particular sites: for example, respiratory, gastrointestinal and haematological cancers have been associated with low cholesterol concentrations in observational studies [1,2]. Substantial numbers of respiratory, gastrointestinal and genitourinary cancers were reported during HPS, so it provides a reasonably reliable (and unbiased) assessment of whether lowering cholesterol by more than 1 mmol/L for about 5 years affects the risks of such cancers. There was no suggestion of any excess in the incidence of cancers at these sites, either overall (table 3) or separately among participants who presented at younger or older age or with lower or higher cholesterol concentrations (figure 11). Even in a study of this size, however, too few cancers occurred at some particular sites (e.g. haematological cancers) for reliable assessment, but an on-going meta-analysis of individual patient data from all major statin trials should be able to provide such information [35].

Individuals with a history of non-melanoma skin cancer remained eligible for inclusion in HPS (although those with a history of other cancers did not), and non-melanoma skin cancer reported following randomisation was to be considered separately from other cancers. During the 5-year scheduled treatment period, there were more reports of non-melanoma skin cancer among the simvastatin-allocated participants (243 [2.4%] vs 202 [2.0%]: RR 1.20 [0.99–1.44]), only one of which was associated with death. This difference is not conventionally significant (p = 0.06), even before allowing for the multiple comparisons involved. Moreover, it did not appear to reflect a clear excess in either basal cell carcinomas (124 vs 99; RR 1.24 [0.96–1.61]; p = 0.1) or in squamous cell carcinomas (137 vs 122; RR 1.12 [0.87–1.42]; p = 0.4). Nor did this slight excess of non-melanoma skin cancer become more apparent with more prolonged treatment, as might be expected if it was causally related. Indeed, the excess was observed largely during years 1 & 2 (107 [1.0%] vs 76 [0.7%]), with little apparent difference during years 3 & 4 (91 [0.9%] vs 86 [0.9%]) or years 5+ (45 [0.5%] vs 40 [0.4%]).

## Discussion

The large size of the Heart Protection Study, its prospective randomised design and the inclusion of a broad range of participants allow it to assess reliably both the efficacy and the safety of cholesterol lowering in a variety of different circumstances. The present results demonstrate that lowering LDL cholesterol by about 1 mmol/L (38 mg/dL) for 5 years with a statin reduces the rate of death from vascular causes by about one-sixth, with no apparent adverse effect on non-vascular mortality or morbidity. This proportional reduction in vascular mortality was remarkably consistent among the different types of participant studied, including women as well as men, older as well as younger individuals, and those who entered the study with below average cholesterol concentrations. Furthermore, the lack of any significant hazard was also consistent among the different types of participant, and there was no suggestion of any adverse effects emerging with more prolonged follow-up.

### Consistent beneficial effects of cholesterol lowering on vascular mortality and morbidity

The prespecified analyses for assessing the benefits of statin allocation in different types of participant were to be of major vascular and coronary events. Because much larger numbers suffered at least one such event, analyses of those outcomes can help interpret the observed effects on the smaller numbers of deaths from vascular causes. For example, the proportional reduction in vascular mortality observed among the 5082 participating women was very similar to that among the men. But since fewer women took part and their absolute risk of vascular death was somewhat lower, this result did not reach conventional levels of statistical significance on its own (p = 0.08). Even so, the very definite and highly significant reductions in major vascular events observed among the participating women (figure 6) indicate that the reduction in vascular death, and hence in all-cause mortality, among the women is real [36,37]. Similarly, the highly significant reductions in major vascular events in the elderly and those with below average cholesterol concentrations at baseline reinforce the consistent reductions in vascular mortality of about one-sixth in these groups. Given the two-thirds compliance during HPS, actual use of 40 mg simvastatin daily in this population would lower LDL cholesterol by about 1.5 mmol/L (57 mg/dL), and this would probably reduce the vascular death rate by about one quarter. Furthermore, the continued divergence in the vascular death rate during successive years of follow-up (reinforced by the similar effect on major vascular events) suggests that more prolonged statin therapy would produce even larger absolute reductions in vascular mortality.

Despite the proven benefits of statins in people with coronary heart disease, it has been suggested that statins may have adverse effects among people with overt heart failure [31]. Only small numbers of participants died from heart failure during HPS, but simvastatin allocation was associated with a marginally significant reduction in heart failure hospitalisations or deaths during the treatment period. Patients with occlusive arterial disease or diabetes who had coexistent heart failure were still eligible for the study provided they were not breathless at rest. Although the presence of heart failure at study entry was not routinely recorded, ACE inhibitors were chiefly used for heart failure or hypertension during the recruitment period (1994–7), which predated evidence from the HOPE trial of benefit in other circumstances [38]. Among participants using ACE inhibitors at baseline, many of whom were likely to have had heart failure, simvastatin allocation significantly reduced the risk of major vascular events (495 [24.9%] simvastatin vs 568 [28.5%] placebo; RR 0.84 [0.75–0.95]; p = 0.006), and there was a non-significant trend towards fewer vascular deaths (265 [13.3%] vs 285 [14.3%]; RR 0.93 [0.78–1.09]; p = 0.4). These results are not consistent with any substantial adverse effect of statin therapy on heart failure, and suggest that the beneficial effect on major vascular events is likely to outweigh any small adverse effect that might exist. Subsequent analyses of HPS based on baseline blood levels of brain natriuretic peptide (which is a sensitive and validated marker of heart failure [39]) should help further to assess the effects of statin treatment in patients with heart failure (as will on-going trials in such individuals [40]).

The significant 13% (SE 4) reduction in all-cause mortality observed in HPS reflects the combined impact in this high risk population of the significant 17% (4) reduction in vascular mortality produced by lowering LDL cholesterol by 1 mmol/L and the lack of any significant effect on deaths from non-vascular causes during 5 years of treatment. Since the proportion of vascular to non-vascular deaths will differ between populations, the observed reduction in all-cause mortality is not readily generalisable to other situations, whereas the reduction in vascular mortality may well be. Direct assessment of the effect of cholesterol lowering on total mortality could also obscure potentially important differences in the effects on cause-specific mortality in particular circumstances [41]. Consequently, the separate analyses of vascular and non-vascular mortality (and morbidity) presented here provide both more sensitive and more generalisable evidence, not only of any beneficial effects of cholesterol-lowering statin treatment but also of any hazards in particular circumstances.

### No evidence of adverse effects of cholesterol-lowering on non-vascular mortality or morbidity

HPS involved much larger numbers of non-vascular deaths, cancers and other serious non-vascular outcomes than any previous cholesterol-lowering study, as well as including large numbers of participants with relatively low cholesterol concentrations. It can, therefore, help address remaining uncertainty as to whether associations in non-randomised observational studies between lower total cholesterol concentrations (e.g. <4 mmol/L) and higher rates of particular non-vascular conditions are causal or, instead, due to confounding or reverse causation [3,4]. Reassuringly, among the 20,000 participants in HPS, lowering total cholesterol by an average of 1.2 mmol/L (46 mg/dL) for 5 years did not appear to have any adverse effect on non-vascular mortality or morbidity. Although there were too few deaths from some particular causes to assess the effects of cholesterol-lowering directly, the results for relevant non-fatal outcomes were of help in overcoming these limitations. For example, despite inverse associations in observational studies between cholesterol concentrations and respiratory mortality [1,2], the randomised evidence from HPS does not indicate any adverse effect of lowering cholesterol on either respiratory mortality or on the much larger number of hospitalisations for fatal or non-fatal respiratory illness (or, indeed, on lung function). Similarly, the apparent lack of any adverse effect in HPS for deaths from cancer, haemorrhagic stroke, liver or renal disease is reinforced by the results for the larger numbers of related non-fatal outcomes.

Some non-randomised observational studies have found lower cholesterol concentrations to be associated with higher mortality in the elderly [32,33], and there has been uncertainty about the effectiveness of cholesterol-lowering in older people [37]. In HPS, however, allocation to cholesterol-lowering statin therapy not only reduced vascular (and total) mortality among 5806 participants aged 70 or over at presentation, but there was also no apparent increase in non-vascular mortality. More recently, a randomised trial reported a marginally significant excess with statin allocation among the 444 patients aged 70–82 at presentation who developed cancer during 3 years of treatment [34]. But that result may well represent a chance finding, since no significant excess was seen with statin allocation among the 674 HPS participants aged at least 70 at presentation who developed cancer during 5 years follow-up, nor has any such excess been reported from the other large statin trials [23]. Chance may also explain the slight (but non-significant) excess of non-melanoma skin cancers observed during HPS, since that excess was largely confined to the early years of the study, was seen with both squamous and basal cell carcinomas, and is not supported by published data from other major statin trials excluding HPS (724 [4.2%] statin vs 699 [4.1%] placebo; odds ratio 1.04; 95% CI 0.93–1.15; p = 0.5) [12,17,22,42].

If lowering cholesterol really did have adverse effects on non-vascular mortality or morbidity [43] then this might be more apparent either among participants who entered HPS with below average cholesterol levels (and so had their cholesterol concentration reduced to very low levels) or after more prolonged exposure to treatment during the later years of follow-up. But even among the 4072 participants whose pretreatment total cholesterol was below 5.0 mmol/L, lowering total cholesterol to an average of 3.5 mmol/L (133 mg/dL) for 5 years was not associated with any excess of non-vascular mortality or morbidity. Nor did any adverse effects begin to emerge with more prolonged treatment and follow-up during the trial. Nevertheless, cancer and other risks may take many years to become manifest, and extended follow-up for mortality and morbidity in HPS (as well as in some other statin trials [44]) will help assess any longer term effects.

### Efficacy and safety of large cholesterol reductions

As might be expected from the approximately log-linear association in observational studies between vascular disease risk and cholesterol concentrations [2,5], a 1 mmol/L LDL cholesterol reduction in HPS from about 4 mmol/L to about 3 mmol/L (i.e. about 155 to about 116 mg/dL) reduced the risks of vascular death by about one sixth and of other major vascular events by about one-quarter, and so too did reducing it from about 3 mmol/L to about 2 mmol/L (i.e. about 116 to about 77 mg/dL), without any evidence of adverse effects. Recently, a randomised study of more intensive statin treatment versus a standard regimen in 4162 patients found that lowering LDL cholesterol to 1.6 mmol/L [62 mg/dL] rather than to 2.5 mmol/L [95 mg/dL] for about 18 months produced a 16% (95% CI 5–26) reduction in major vascular events [45]. These findings indicate that any thresholds below which lowering LDL cholesterol does not safely reduce vascular disease risk are at much lower concentrations (e.g. below 2 mmol/L [77 mg/dL] of LDL cholesterol or 3.5 mmol/L [135 mg/dL] of total cholesterol) than are typically seen in Western populations. Several large-scale trials that are currently assessing more intensive statin regimens [46-48] will provide further information as to whether even more substantial cholesterol reductions are not only effective at lowering vascular disease risk but also safe.

## Conclusion

Based on large numbers of deaths and other relevant outcomes, the present results show that lowering LDL cholesterol by an average of 1 mmol/L produces substantial reductions in vascular (and, hence, all-cause) mortality in a wide range of individuals at increased risk of occlusive arterial disease (as well as reducing their risks of heart attacks, strokes and revascularisation procedures). These results also provide considerable reassurance that lowering total cholesterol concentrations by more than 1 mmol/L for an average of 5 years does not produce adverse effects on non-vascular mortality or cancer incidence, even among those who had their cholesterol concentrations reduced to very low levels. Indirectly, this observation provides some reassurance about the likely efficacy and safety of the more intensive cholesterol-lowering achievable with higher-dose or newer statins, and with combinations of standard statin doses and drugs acting through other pathways (such as resins or cholesterol absorption inhibitors). The present results provide further evidence of the benefits and safety of using statin therapy routinely in anyone (irrespective of their initial cholesterol concentration or other factors, such as age or gender) in whom a reduction in their vascular disease risk of about one third would be considered worthwhile.

## Competing interests

The Clinical Trial Service Unit has a staff policy of not accepting honoraria or other payments from the pharmaceutical industry, except for the reimbursement of costs to participate in scientific meetings. Coordinating centre members of the writing committee (J Armitage, R Collins, L Bowman, S Parish, R Peto) have, therefore, only had such costs reimbursed. P Sleight has received honoraria as well as such reimbursement of costs.

## Authors' contributions

MRC/BHF Heart Protection Study Collaborative Group

*Writing Committee*-Jane Armitage, Rory Collins, Louise Bowman, Sarah Parish, Peter Sleight and Richard Peto.

JA was involved in data collection, analysis and interpretation, and drafted the manuscript; RC conceived and designed the study and was involved in data collection, analysis and interpretation, and drafting of the manuscript; LB was involved in data collection and drafting of the manuscript; SP was involved in data collection, analysis, validation and interpretation; RP conceived and designed the study, and was involved in analysis and interpretation; PS was involved in the study design and interpretation. All authors provided comments on and approved the final manuscript.

*Steering Committee*-R Collins (principal investigator), T Meade (chairman), P Sleight (vice-chairman), J Armitage (clinical coordinator), S Parish and R Peto (statisticians), L Youngman (laboratory director), M Buxton, D de Bono (deceased), C George, J Fuller, A Keech, A Mansfield, B Pentecost, D Simpson, C Warlow; J McNamara and L O'Toole (MRC observers).

*Data Monitoring Committee*-R Doll (chairman), L Wilhelmsen (vice-chairman), K M Fox, C Hill, P Sandercock.

*Collaborators*- *(doctors; nurses; receptionists): Aberdeen Royal: *N Benjamin, J Webster; J Jamieson; L Donald. *Bassetlaw Hospital: *R Blandford; L Carrington, H McMahon; D Cheetham. *Royal United, Bath: *J Reckless; L Brice, R Carpenter, J Christmas; C Flower. *Bedford: *I Cooper; S Frampton, E Pickerell; J Wells. *Belfast City: *M Scott; V Crowe, A Shaw; L Shannon. *Birmingham City: *S Jones; G Faulkner, A Lavery, H O'Leary, R Watson; C Capewell, S Hughes. *Birmingham Heartlands: *S Bain, A Jones; G Holmes, C Jewkes; T Bellamy, P Harrison. *Queen Elizabeth, Birmingham: *N Buller; J Hooks, H Jones, E Smith, P Vint, R Watson; P Crook, J Williams. *Bishop Auckland General: *M Bateson; P Cawley, P Gill; L Hawkeswell, K Simpson. *Royal Bournemouth: *M Armitage; C Cope, J Tricksey, M Wilson; S Cottrell. *Princess of Wales, Bridgend: *C Jones; M Llewellyn, P Smith; T Woodsford. *Royal Sussex County, Brighton: *R Vincent; E Joyce, N Skipper; P Peters. *Bristol Royal Infirmary: *M Lemon (late), D Stansbie; A Hagos Kidan, M Halestap; A Gibbons, J Meredith. *Frenchay, Bristol: *C Dawkins, M Papouchado; L Baker, K Boulton, C Dawe; A Lewis, J Wisby. *Addenbrooke's, Cambridge: *M Brown; J Emeny, W Smith, D Thurston, D Trutwein; M Cornwell, D Lloyd. *Castleford & Normanton: *C White; M Hudson, M Khalifa, N MacKereth, J Woolford; G Martin. *St Peter's, Chertsey: *M Baxter; R Chambers, S Glenn, J Kerr; G Golesworthy, A Watts. *Corby Community: *G Baines; J Groom, L Price; I Barlow. *Leighton, Crewe: *S Mallya; J Maiden, M Nash; V Lowe. *Derbyshire Royal Infirmary: *M Millar-Craig, A Scott; S Cozens, J Hannah, M Hinwood, S. Hopcroft, M Margetts, H Waterhouse; J Millward. *Darlington Memorial: *J Murphy; M Charters, B Graham; M Banks, M Boon, C Cassidy, R Nobbs, *Dewsbury District: *T Kemp; P Turner; S Sheldrake. *Russells Hall, Dudley: *M Labib; R Pearson, J Sidaway; P Davies, M Hodgkiss. *Queen Margaret, Dunfermline: *D MacLeod; R Stuart; J Albrock, J Fisher, F Stuart. *Edinburgh Royal Infirmary: *C Swainson; S Glenn, J Johnston, S Sadler; M Curren, S Feirnie, L Stenhouse. *Western General, Edinburgh: *R Lindley, C Warlow; A Kenny, F Waddell; M Brownlie, I Guilar. *Derriford, Plymouth: *A Marshall, J Went; S Clarke, A Inman, J Simmonds; B Duook, G Mortimore, A Pascoe. *Glasgow Royal Infirmary: *S Cobbe; C Campbell, H Young; M Keeble. *James Paget, Great Yarmouth: *S Absalom; L Baillie, N Bracey; L Falco, D Stone. *Hartlepool General: *G Tildesley; B Carr, G Longstaff, A Turner, H Wilkinson; S Wilkinson. *Hillingdon: *R Hillson; D Brookes, B Capper, N. Mahabir, K Price; V Badrick. *Huddersfield Royal Infirmary: *H Griffiths; J Fitzgerald, S Lewis; P Campbell. *Kettering General: *G Baines; J Cullen, G Claypole, J Lomas; A Rogers. *Royal Lancaster Infirmary: *A Brown; J Cheshire; J Rowley. *Leeds General Infirmary: *S Ball, C Prentice, A Hall; P Atha, K Caffrey, W Currie, K Drury, C Hague, S Hall, P Maguire, C Rose, R Watson; A Buxton, A Wedgwood. *St James University, Leeds: *S Gilbey; W Currie, K Drury, S Hall, C Rose, J Wilson; M Vaughan. *Walton Centre, Liverpool: *P Humphrey; J Blocksage, R McSloy, K Ost, L Owen, S Saminaden, D Watling, J Wiseman; J Davies. *Ealing General, London: *A Kehely, J Kooner; I Corbett, J Peters, K Price, S Trainor; M Van Goethem. *Guy's & St Thomas', London: *J Chambers; M Crawshaw, A Jones, J O'Sullivan, S Powell, M Reoch, J Sanders; M-F Beament, B Fangrad, Y Williams. *North Middlesex, London: *S Banim, T Crake; B Ford, V Glynn; S Ismail. *Royal Brompton, London: *N Buller, A Coats; L Aitken, E Cruddas, K Serup-Hansen; D Nosworthy, N Reilly. *Whittington, London: *S Coppack, J Malone-Lee; P Clifton, A Holmes; L Camplin. *Luton & Dunstable: *D Peterson, C Travill; S Gent, A Hunter, C Stroud; K Griffiths. *Macclesfield District General: *E Davies; M Mason, A Robinson; S Belfield. *Maidstone: *J Chambers; L Bispham, J Massey, A Mercer, J Sheppard; S Burrage. *Manchester Diabetes Centre: *K Cruickshank; KL Chan, V Wharfe, J Woodward; F Alexander, Y Williams. *Manchester Royal Infirmary: *M Walker; P Campbell, J Day, S Edwards, B Kelly, P Nicholson; S Barrett, S Gleeson. *North Manchester General: *M Savage, J Swan; D McSorland, Gillian Thompson, C Waywell; C O'Neill, L Wharton. *Royal Victoria Infirmary, Newcastle-upon-Tyne: *P Adams, R Lindley, N Cartilidge; M Mace, M Thompson; J Hulmes. *Radcliffe Infirmary/John Radcliffe, Oxford: *J Armitage, R Collins, P Sleight; S Beebe, M Campbell, J Godden, S Goodwin, A Lawson, H Lochhead, P Whitbread; S Knight, A Taylor, S Turner; *Royal Alexandra, Paisley: *I Findlay; C Campbell, J Hunter, H Young; E McNally. *Whiteabbey, Newtownabbey: *P Crowe; V Crowe, B Hunter, A Shaw; L Shannon. *North Tyneside Health Care Centre, North Shields: *R Curless, R Lindley, P McKenna, S Roberts; A Black, J Martin; M Burt. *Northampton General: *J O'Donnell; T Burdett, S Marsh, J Woodward; R O'Hare, C Owen. *Poole General: *A McLeod; M Richardson; C Reeves. *Halton General, Runcorn: *R Mallya; J Forshaw, J Hodson; H Lenden, G Osborn. *St Helier: *J Barron; A Ballard, B Docherty, M McDonnell, S Ritson, D Tyler; S Carter, C Rigney. *Conquest, St Leonard's-on-Sea: *R Wray; K Gaughan, J Sinclair; J Burleigh, J MacDonald. *Royal Hallamshire, Sheffield: *G Venables; C Doyle, M Fox, L Mundey; D Thompson, S Rowley. *King's Mill Centre, Sutton-in-Ashfield: *R Lloyd-Mostyn; D Bailey, I McKenzie; R Bamford. *Singleton, Swansea: *P Thomas; R Thomas; C Alexander, R Chohan, K Wood. *Princess Royal, Telford: *N Capps; D Donaldson, C Stiles, L Tonks; S Crank. *Manor, Walsall: *A Cunnington, P Giles; N Groves, E Walton; W Dance. *Watford General: *M Clements; C Feben, A Hunter, E Walker; L Atkins, R Kaiser, R Williats. *Sandwell District General, West Bromwich: *E Hughes; J Elson-Whitaker, S Sumara, C. Verow; G Banks, R Glover, K Hall. *Worcester Royal Infirmary: *A Munro, C Pycock, D Tibbutt; J Cadwell, M Greenwood; M Betts. *Worthing: *M Signy; E Joyce, C Wrapson; G McCourt, R Moore. *Wycombe General: *S Price, R Regan; M Aldersley; P Pendry. *Coordinating Centre administration and computing, Clinical Trial Service Unit, University of Oxford: *J Barton, C Bray, and K Jayne (administrative coordinators), V Booker, H Bojowsky, R Brooker, M Corbett, J Crowther, A Grantham, C Harwood, D Haywood, J Heineman, C Hope, C Indge, R Jones, S Jones, R Kanahan, K Kidney, M King, S Knight, H Lang, C Mardsen, C Mathews, G Meade (deceased), H Monaghan, K Murphy, A Naughton, A Owers, A Peto, S Pickworth, G Pocklington, A Radley, S Southren, K Szumczyk, R Tong, E Wincott; Clinical support and outcome adjudication: J Armitage and R Collins (study coordinators), A Keech and S MacMahon (piloting and planning), C Baigent, L Bowman, K Burbury, Z Chen, R Clarke, S Dunachie, V Frigi, M Landray, E Lau, C Sudlow, C Turnbull; Statistics and computing: S Parish and R Peto (statisticians), P Harding, M Lay, and K Wallendszus (computing coordinators), N Bruce, A Charles, A Cody, N Goodwin, R Greenlaw, B Hauer, P McCabe, A Palmer, C Peto, A Rowe, S Wilson, A Young, A Young; *Coordinating Centre laboratory, Clinical Trial Service Unit, University of Oxford: *L Youngman (director), K Kourellias, S Clark, and M Radley (laboratory coordinators), K Bhamra, L Buckingham, M Bradley, T Chavagnon, B Chukwarah, C Colominas, S Crowley, K Emmens, S Edwards, J Gordon, J Hill, A Lee, C Lennon, M McAteer, N Miller, S Norris, H Priestley, J Taylor, J Wintour, M Yeung; *Nurse Monitors and trainers: *S Beebe, M Campbell, J Fitzgerald, J Godden, A Lawson, S Lewis, H Lochhead, M McDonnell, M Nash, P Whitbread.

## Pre-publication history

The pre-publication history for this paper can be accessed here:


